# Novel and Previously Known Mutations of the *KCNV2* Gene Cause Various Variants of the Clinical Course of Cone Dystrophy with Supernormal Rod Response in Children

**DOI:** 10.3390/jcm13164592

**Published:** 2024-08-06

**Authors:** Almaqdad Alsalloum, Ilya Mosin, Kristina Shefer, Natalia Mingaleva, Alexander Kim, Sofya Feoktistova, Boris Malyugin, Ernest Boiko, Shamil Sultanov, Olga Mityaeva, Pavel Volchkov

**Affiliations:** 1 Federal Research Center for Innovator and Emerging Biomedical and Pharmaceutical Technologies, 125315 Moscow, Russiavpwwww@gmail.com (P.V.); 2 Pediatric City Clinical Hospital Named for Z.A. Bashlyaevoy, 129272 Moscow, Russia; 3 S. Fyodorov “Eye Microsurgery” Federal State Institution St. Petersburg Branch, 192283 St. Petersburg, Russia; 4 S.N. Fedorov National Ophthalmology Medical Research Center “Eye Microsurgery”, 127486 Moscow, Russia; 5 North-Western State Medical University Named after I.I. Mechnikov, 191015 St. Petersburg, Russia; 6 Artema Therapeutics Inc., Brooklyn, NY 11201, USA; 7 Department of Fundamental Medicine, Lomonosov Moscow State University, 119992 Moscow, Russia; 8 Moscow Clinical Scientific Center N.A. A.S. Loginov, 111123 Moscow, Russia

**Keywords:** cone dystrophy, *KCNV2*, electroretinogram, compound heterozygous mutations, genetics

## Abstract

**Background/Objectives**: Cone dystrophy with supernormal rod response (CDSRR) is a rare autosomal recessive retinal disorder characterized by a delayed and markedly decreased photoreceptor response. In this article, we aim to describe the clinical course and associated molecular findings in children with cone dystrophy with supernormal rod response associated with recessive mutations in the *KCNV2* gene, which encodes a subunit (Kv8.2) of the voltage-gated potassium channel. **Methods**: The genetic testing of two patients included the next-generation sequencing of a retinal dystrophy panel and direct Sanger sequencing to confirm *KCNV2* gene variants, in addition to an electroretinogram (ERG) and spectral domain optical coherence tomography (SD-OCT). **Results**: Cone dystrophy with supernormal rod response is associated with identified variants in the *KCNV2* gene. The genetic analysis of the first case identified a compound heterozygous mutation in the *KCNV2* gene, including a de novo nonsense duplication at cDNA position 1109, which led to the premature termination of the p.Lys371Ter codon in the second extracellular domain of the protein. Two patients showed changes in the full-field electroretinogram, especially in the first case, which demonstrated a close to supernormal total electroretinogram amplitude. This study increased the range of the *KCNV2* mutation database, added an unreported de novo substitution pattern to *KCNV2* gene variants, and linked it to the evaluated clinical studies. **Conclusions**: The initial clinical manifestations were varied, but both patients presented with hypermetropia and slight exotropia. The ERG findings are characteristic of *KCNV2* mutations, and patients exhibited an increased b-wave latency in DA3.0 ERG (combined rod–cone response).

## 1. Introduction

Cone dystrophy with a supernormal rod response (CDSRR; OMIM # 610356) is a rare, inherited, retinal disorder related to mutations in the potassium voltage-gated channel modifier subfamily V member 2 (*KCNV2*) gene. CDSRR can be inherited due to autosomal recessive form mutations in the *KCNV2* gene on chromosome 9 [1,2]. The associated symptoms appear in the first two decades of life [3]. Furthermore, patients with CDSRR may experience nyctalopia, photophobia, and mild to moderate myopia until the second decade of life as a result of changes in the macular retinal epithelium and retinal structure and function. Cases of CDSRR are most often reported in consanguineous marriages, which may increase the risk of recessive diseases. CDSRR was first described by Gouras et al. in 1983, when it was described in a Hispanic family. Since then, CDSRR has been described as a rare retinal disorder with autosomal recessive inheritance; it is also associated with suprathreshold rod responses and significant reductions in cone–rod electrophysiology (ERG) under high-stimulus conditions [4,5,6].

Childhood-onset CDSRR is characterized by a significant reduction in visual acuity, typically reaching 20/100 or worse during the second decade of life. The majority of affected individuals exhibits myopia and a delayed onset of night blindness. Observations indicate that certain patients with CDSRR may present with a normal fundus, accompanied by parafoveal or foveal atrophy. Additionally, macular bull’s eye patterns, hyperfluorescence anomalies, and generalized fine pigmentary retinopathy have been documented in the literature [7,8]. The prevalence of CDSRR in the USA is estimated to be approximately 1 in 865,000, thereby categorizing the condition as rare. The advent of electroretinography (ERG) and genetic testing has enabled the precise and timely diagnosis of CDSRR, thereby facilitating the advancement of therapeutic interventions for this condition [4,9,10,11].

Pathogenic variants in the *KCNV2* gene can affect the Kv8.2 members of voltage-gated potassium channels (Kv channels) [1]. Kv8.2 congregates with Kv2.1, forming a heteromeric type of Kv channel, which is predominantly expressed in retinal rod and cone photoreceptors. Substitutions in the *KCNV2* gene have been suggested to present disorders in the morphology and function of Kv8.2. Furthermore, mutations in the *KCNV2* gene could affect the steady activation current Ikx, followed by alterations in the total potential of the photoreceptors’ membrane. Nevertheless, substitutions may lead to the inhibition of the favorable assembly of heteromeric voltage-gated potassium channels during disturbances in the photoreceptor membrane potential, caused by long-term external potassium currents in dark conditions [12]. The electroretinograms of patients with CDSRR have a special response pattern. Cone photoreceptor responses may show a delay and decrease in dim flash, and rod responses have b-wave changes that show subnormal activity. However, at a high stimulation intensity, the responses of photoreceptor rods predominantly have a normal or increased amplitude [2].

Our study reports cases of compound heterozygous variants of the *KCNV2* gene and patients with CDSRR, including a de novo mutation type (c.1109dup) in the first case described, accompanied by a description of their phenotypes.

## 2. Materials and Methods

### 2.1. Clinical Assessment

Clinical observations for the patients were obtained from inherited retinal disease specialists, including detailed history, best-corrected visual acuity (BCVA) in logMAR scale (Topcon-CC-100, The Netherlands), slit-lamp biomicroscopy findings (Slit lamps Takagi, Nagano, Japan), fundoscopy, retinoscopy (HEINE Optotechnik, Gilching, Germany), short-wave blue fundus autofluorescence (FAF), and spectral domain optical coherence tomography (SD-OCT) (DRI OCT Triton, Topcon Healthcare, The Netherland).

### 2.2. Electrophysiological Assessment

A full-field electroretinogram (ffERG) recording (Tomey EP1000, Japan) was conducted according to the recommendations of the International Society of Clinical Electrophysiology of Vision (ISCEV) [12]. The used protocol included a dark-adapted (DA) 0.01 electroretinogram (ERG) (rod response), DA 3.0 ERG (combined rod–cone response), light-adapted (LA) 3.0 ERG (cone response), and LA 3.0 ERG (30 Hz flicker).

### 2.3. Molecular Genetics

The patients underwent next-generation sequencing via the retinal dystrophies panel at the Research Centre for Medical Genetics in Moscow, Russia. Genomic DNA was extracted from the patients and their families using the ExtractDNA Blood Kit (Evrogen, Russia) according to the manufacturer’s instructions. The *KCNV2* gene was PCR amplified and subsequently, Sanger sequencing using the following pairs of primers was performed: GTGCGACGACTACGAGGAGCA/CGAGCGTGAAGAAGCCCATGCA and GTGGAGGAGATGCAGCAGCA/GGAgTGGGGGATGGTAGTGAA, while we used the pair of primers GTGAGGCCATCAGCAATA/ATACAAGGACTTCATGAT to detect the *IMPG2* gene. The Genome Aggregation Database was used as a reference dataset to estimate the minor allele frequencies for identified mutations in the general population http://gnomad.broadinstitute.org (accessed on 3 August 2024).

## 3. Results

### 3.1. Subjects and Clinical Findings

This article highlights two cases involving *KCNV2* associated with retinopathy characterized by significant changes in overall electroretinogram results and a decrease in the thickness of the retinal neuroepithelium. After clinical observations and molecular analysis, the patients were found to have mutations in the *KCNV2* gene, both of which were compound heterozygous. One of them was found to have a de novo heterozygous mutation, which could lead to slight changes in a patient’s phenotype and significant alterations in visual function [13].

**Figure 1:**
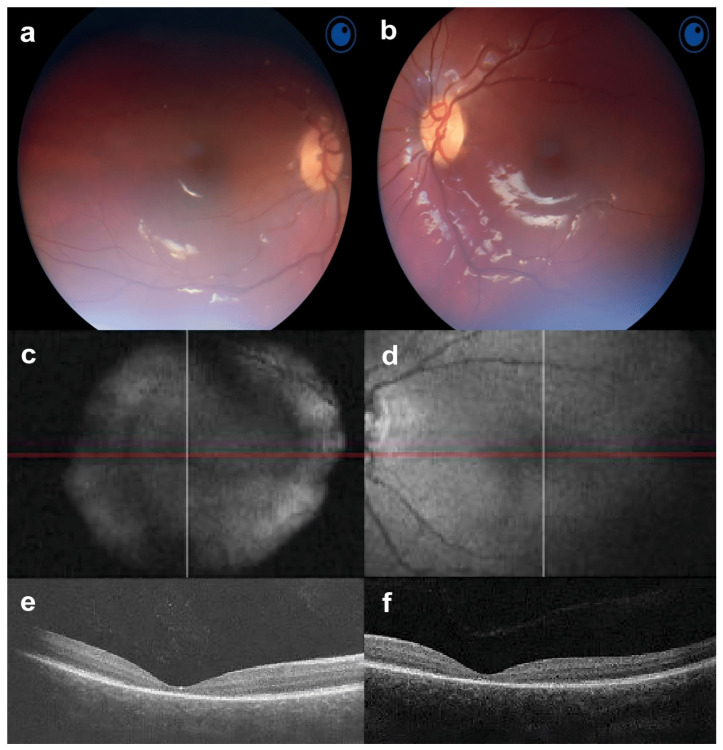
Fundus photos, fundus autofluorescence (FAF) and optical coherence tomography (OCT) of the first case in both eyes. (**a**,**b**) Fundusoscopy shows that the optic disc is pink, with clear contours and normal retinal vessels; (**c**,**d**) FAF shows an enlarged macular reflex that is somewhat darkened and blurred; (**e**,**f**) OCT shows the perspicuous thinning of the retina in all layers, and is more pronounced in the foveal zone.

The first clinical case features a 7-year-old girl with a *KCNV2* gene mutation. The patient’s visual problems began in childhood. At the age of three months, she was diagnosed with hypermetropia. At the age of 4 months, nystagmus began to be observed, especially while focusing on objects. Subsequently, the nystagmus persisted, but decreased slowly and became barely noticeable in one eye. The girl was born full term and her mother had a healthy pregnancy. The parents do not have any vision anomalies. There was neither a consanguineous marriage nor a family history of documented retinal disease. At the age of one year, it was noticed that the patient could constantly look at light directly without pain and had a hard time focusing. At the age of three years, BCVA 0.7 logMAR was observed in both OD and OS, she also had mild hypermetropia and latent nystagmus. A full electrophysiological examination at the age of 5 years revealed changes in the retina, which cast doubt on gene-related retinopathy. The electroretinogram (ERG) was abnormal in the main rhythm. Her last examination at the age of 6 years did not reveal any changes in the anterior segment of the eye or the vitreous body. On ophthalmoscopy, the optic disc was normal with clear lines and a deposition of pigment along the contour. Normal retinal vessels were observed (Figure 1a–d). Macular reflex was enlarged, and somewhat obscured and blurred. The macula had dyspigmentation and the peripheral retina was without pathology. BCVA was 1.0 logMAR in OU, and she was diagnosed with a complex hypermetropic astigmatism, latent nystagmus, and concomitant divergent alternating intermittent strabismus. Optical coherence tomography (OCT) revealed a significant thinning of the retinal neuroepithelium in all layers, more pronounced in the foveolar zone. The average thicknesses of the neuroepithelium in the fovea were 131 μm and 120 μm for the right and left eyes, respectively. At the same time, the thickness of the retinal nerve fiber layer corresponded to the average for her age (110.9 + 9.7 µm)—the overall average thickness was 85 µm on the right and 87 µm on the left (Figure 1e,f). The proband’s electrophysiological details were observed and the total electroretinogram amplitude was normal (even close to supernormal). For the DA3.0 ERG, a latency in a and b-waves increased. The oscillatory potentials were abnormal. The LA3.0 ERG was sharply subnormal with a 80–90% reduction in a-b-wave amplitudes and an increasing latency of a- and b waves. Rhythmic ERG (30 Hz) was lower than a normal amplitude.

**Figure 2:**
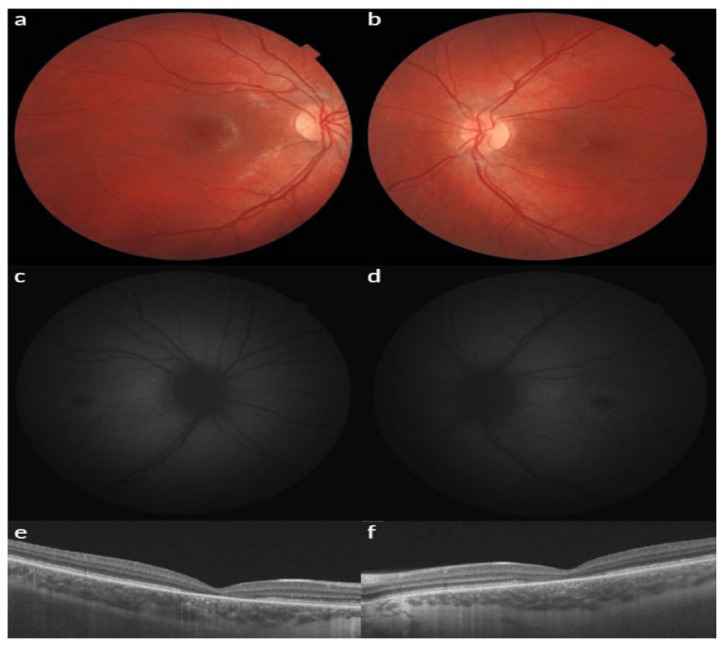
Fundus photos, fundus autofluorescence (FAF) and optical coherence tomography (OCT) of the second case in both eyes. (**a**,**b**) The last fundoscopy reveals a standard optic disc and retinal vessel morphology with macular and foveal reflexes absent. (**c**,**d**) FAF presents symmetrical abnormal spots of hypoautofluorescence surrounded by a ring of hyperautofluorescence; (**e**,**f**) OCT shows a slight decrease in the average thickness of the neuroepithelium with the absence of the myoid and ellipsoid zones of the fovea.

**Figure 3:**
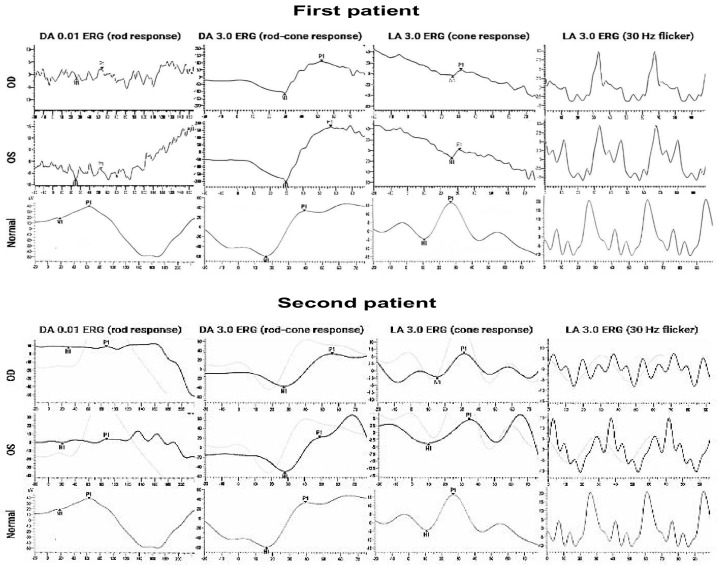
Full−field ERG in the first and second cases is comparable with a normal ERG. The first patient ERG was close to supernormal. The scotopic rod–cone response revealed an increase in the latency of the a− and b− waves. ERG is subnormal; the amplitudes of a–b waves are reduced. The flicker response amplitude was found to be reduced compared to the norm. The second patient’s ERG revealed a decrease in the amplitude and an increase in the latent period of the b-wave in both cone and rod responses. The amplitude of the flicker ERG response decreased compared to the age norm.

At the first examination of a 4-year-old boy (the second case), BCVA was 1.0 logMAR for both OD and OS. In addition, photophobia and hypermetropia of +1.5 diopters were diagnosed. According to the Hirschberg test, the boy had exotropia up to 10°; there were no changes in the anterior segment of the eye or the vitreous body. A fundoscopy observation revealed a standard optic disc and retinal vessel morphology (Figure 2a,b). There were no changes in the peripheral retina, while macular and foveolar reflexes were absent. According to the FAF, symmetrical abnormalities in the form of a rounded focus of hypoautofluorescence surrounded by a ring of hyperautofluorescence were noticed (Figure 2c,d). A noticeable decrease in the thickness of the neuroepithelial in the fovea was revealed; the average thicknesses in the macula were 166 μm and 169 μm for the right and left eyes, respectively. Time domain OCT also revealed the absence of a highly reflective band associated with the reflection of the conjunction of the outer–inner segment (IS/OS) of the photoreceptors. The overall average thickness of the nerve fiber layer corresponded to the age norm: 111 µm on the right, 112 µm on the left. ERG showed a decrease in the amplitude of the maximum in the cones, and the b-wave of the rod response was close to the lower limit of the normal ERG. Furthermore, a 12–15 ms prolongation of b-wave latency was observed. At the age of 8 years, an examination revealed that visual acuity had not changed, but photophobia and color vision disturbances were noted. Automatic static suprathreshold perimetry showed decreased foveal photosensitivity, an extensive central absolute scotoma, and multiple relative paracentral and peripheral scotomas. Spectral OCT data revealed a slight decrease in the average thickness of the neuroepithelium, especially in the fovea. The average neuroepithelial thicknesses were 241 µm and 255 µm for the right and left eyes, respectively. In addition to the absence of the highly reflective band, the fovea lacked myoid and ellipsoid zones. The overall average thicknesses of the retinal nerve fibers were 118 µm on the right and 120 µm on the left, which is comparable to the age norm (Figure 2e,f). Repeated ERG recording at 8 years of age found a significant decrease in the amplitude and prolongation of the latent period of the b-wave in cone and rod responses. Likewise, a decrease in the amplitude of oscillatory potentials and rhythmic ERG was observed (Figure 3).

### 3.2. Molecular Genetics

Two patients were subjected to next-generation sequencing via the retinal dystrophies panel and direct sequencing of the *KCNV2* genome to identify pathogenic variants.

**Figure 4:**
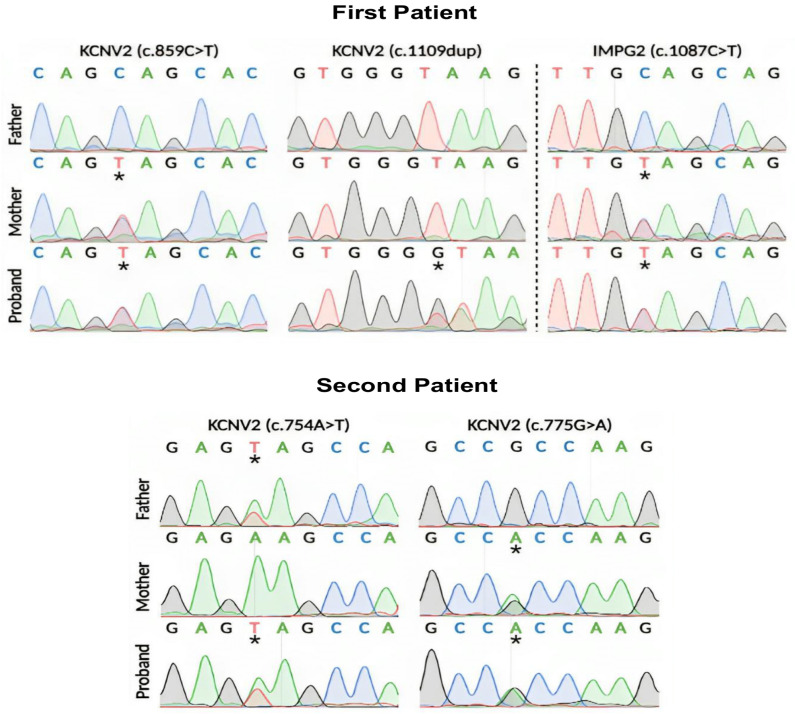
Direct sequencing of the *KCNV2* gene in the first and second patients. The first patient revealed a de novo mutation, c.1109dup, and another at position c.859C>T, forming a complex heterozygous mutation in the proband. The second patient revealed a complex heterozygous mutation, containing c.754A>T and c.775G>A substitutions. Substitutions are marked with asterisks.

The first case involves a compound heterozygous mutation in the *KCNV2* gene (Figure 4). The first substitution is a C>T in cDNA position 859, which leads to a premature termination codon, p.Gln287Ter, in the protein’s first extracellular domain. The previous nonsense mutation is present in the population database (GnomAD exomes, number of homozygous alleles = 0) at an exceedingly low frequency (8:627,570 alleles, 0.00001275), the variant affecting the *KCNV2* gene, which is associated with retinal cone dystrophy type 3B (610,356; AR). A previously undescribed second variant in a heterozygous state was detected in the first exon (of two) of the *KCNV2* gene in the first case. A frameshift indel mutation, which is a duplication at cDNA position 1109, would lead to the premature termination of the p.Lys371Ter codon in the second extracellular domain of the protein. However, this gene is not in the GenomeAD population database, nor is it mentioned in the literature. In addition, the mutation is more likely to be pathogenic, and pathogenic variants of the *KCNV2* gene can lead to retinal cone dystrophy type 3B. Since the first patient has a compound heterozygous mutation, the direct analysis of the *KCNV2* gene in the family members of the first proband presumes that the c.859C>T substitution was bequeathed from the mother. The father also underwent a sequencing analysis of the *KCNV2* gene to confirm the source of the duplication variant at cDNA position 1109. However, the proband father’s sequencing was normal and the duplication is absent, which suggests that the c.1109dup is a de novo type of mutation, where the mutation spontaneously occurred and was not inherited from the patient’s parents. Short tandem repeat (STR) analysis was performed on family members to determine an individual’s DNA profile as an additional test to track down and verify family connections. Another mutation in the gene was found in the heterozygous state in exon 10 (out of 19). The previously undescribed occurrence is a C>T in cDNA position 1087 (Figure 4). Seemingly, the variant leads to the appearance of a stop codon and premature termination of protein translation p.Gln363Ter (mutation of nonsense type). The pathogenic biallelic variants in this gene can lead to the development of retinitis pigmentosa type 56 (613581; AR), while the heterozygous form can cause vitelliform macular degeneration type 5 (616152; AD). The variant is present in the population database GenomAD with a frequency of 0.0004%.

The second case is also a compound heterozygous mutation in the *KCNV2* gene (Figure 4), the first substitution being an A>T at cDNA position 754 in the first exon. The substitution variant is believed to terminate the translation of p.Lys252Ter in the N-terminal domain of the protein. The mutation is in a heterozygous state and is present in the population database (GnomAD exomes, number of homozygous alleles = 0) with an extremely low frequency (2:1458724 alleles, 0.000001371). The mutation affecting the *KCNV2* gene is highly probable to be pathogenic, since mutations in this gene have been described in patients with retinal dystrophy type 3B, which is inherited in an autosomal recessive manner. The second proband has a second mutation, which is a G>A substitution at cDNA position 775 in the first exon. The substitution variant is a missense-type mutation and is believed to alter the translation of p.Ala259Thr, which may affect protein structure/function in the N-terminal/first extracellular domains. The heterozygous state of the mutation is present in the population database (GnomAD exomes, number of homozygous alleles = 0) with a low frequency (3:625,134 alleles, 0.000004799). This variant is suspected to be pathogenic and associated with retinal dystrophy type 3B. Substitutions c.775G>A and c.754A>T in the *KCNV2* gene were detected in both maternal and paternal sequencing, respectively.

## 4. Discussion

The present work describes two cases of cone dystrophy with a supernormal rod response (CDSRR). The patients are from two different families showing different retinopathy-associated variants in the *KCNV2* gene. One variant is a de novo type of mutation and is not delineated in previous studies or the disease database ClinVar. The variants also demonstrated a rare heterozygous mutation in the *KCNV2* gene inherited from their parents, except the de novo variant. This study is the first of its kind, describing the clinical and genetic attributes of two patients diagnosed with cone dystrophy with a supernormal rod response (CDSRR) in Caucasian patients in Russia.

In both patients, hypermetropia and slight exotropia were observed in the early onset of CDSRR. In the first case, nystagmus was unstable and was first observed at 4 months of age; subsequently, nystagmus persisted, but decreased and became barely noticeable in one eye. Conversely, other studies mentioned a CDSRR patient with early childhood nystagmus had failed to show any improvement with age, regardless of the fact that the patient was relatively young [14]. Giving consideration to the short follow-up and young age, the patients maintained relatively stable BCVA, since progression in CDSRR at the level of cone dystrophy is not significantly accelerated and patients usually have stable or mild changes in BCVA [5,14]. Pre-eminently, the second case showed photophobia, color vision impairment, and extensive central scotoma, as well as multiple relative paracentral and peripheral scotomas, which are also features of CDSRR patients mentioned in previous studies [2,15].

Macular dyspigmentation in the first case and the symmetrical abnormalities of hyperautofluorescent rings in the second case are considered evidence of RPE defects. Moreover, the round focus surrounded by a ring of hyperautofluorescence found in the FAF of the second patient uncovered the fact that the RPE was being affected and was incapable of processing the remnants being released from the photoreceptors [16]. Mainly, the marked decrease in neuroepithelial thickness in all layers in both patients is likely a consequence of the atrophy in the hyperautofluorescent-encompassed RPE areas, which is considered to be a precursor to photoreceptor death [17,18]. Interestingly, the absence of the highly reflective band was particularly noticed in the central macular region, which is associated with the co-occurrence of the outer and inner segments of the photoreceptors in the second case [19]. Furthermore, the significant thinning of the retinal neuroepithelium pronounced in the foveal zone and the normal peripheral region of the retina in the first case is reminiscent of the fact that Müller cells are absent from the fovea. Müller cells’ absence explains the cones being more endangered, while rod-rich areas maintained their morphological integrity in the peripheral retinal areas [20].

**Figure 5:**
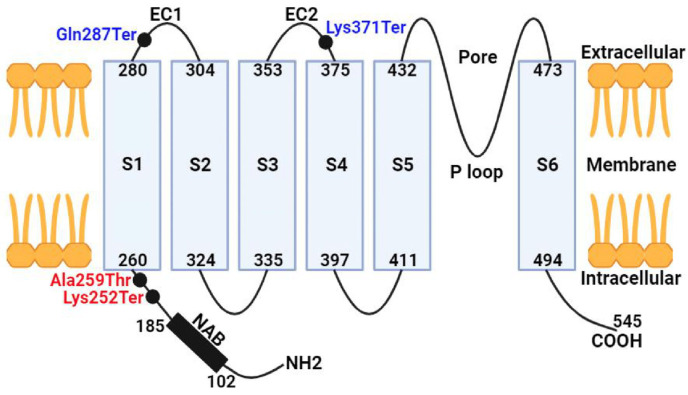
Schematic diagram of the *KCVN2* potassium channel subunit. The structure of the alpha subunit of the potential-dependent potassium channel is represented by N-terminal A- and B-boxes (NABs), transmembrane domains (S1–S6), extracellular domains, and a P loop motif between S5 and S6. The approximate location of mutations (black dots) related to the patients is shown in the diagrammatic structure in blue and red for the first and second cases, respectively.

Direct sequencing in both patients revealed disease-causing mutations forming a compound heterozygous mutation pattern in the *KCNV2* gene. In the first case, the c.859C>T substitution was identified, which was predominantly inherited from the mother, and in the second variant, c.1109dup was identified de novo and does not exist in the parents. However, both previous variants are nonsense mutations, leading to a premature termination codon in the extracellular domain of the protein (Figure 5), resulting in the destruction of the *KCNV2* protein and a non-functional product. The c.859C>T variant was previously mentioned in a case where the patient’s phenotype manifested no nystagmus, but had a color vision deficiency with night blindness, which contrasts our first case where an unstable nystagmus was observed. The difference between cases is likely due to the presence of a second different heterozygous variant in each patient. However, both patients had RPE defects, especially in the foveal region [2].

An *IMPG2* gene substitution was also detected in the first proband and was inherited from the mother (c.1087C>T). The heterozygous form of the *IMPG2* gene in the first case can cause a vitelliform macular dystrophy-associated phenotype in the case of the heterozygous complex allele in *IMPG2* [21], while the biallelic mutation in *IMPG2* can be related to the retinitis pigmentosa, which leads to early macular involvement [22].

In the second case, a heterozygous mutation was also observed in the *KCNV2* gene, composed of c.754A>T, also found when sequencing the paternal *KCNV2* gene, and c.775G>A, inherited from the mother. It is assumed that one of the previous substitutions leads to the formation of a stop codon (c.754A>T) in the N-terminal domain of the protein. Both mutations are predicted to result in a loss of normal protein function, which could be due to protein truncation, nonsense degradation, or even the formation of a nonconducting heteromeric subunit.

The electrophysiological recording in the first patient was likely related to the CDSRR, which was an approximately supernormal total ERG. In addition, the first case showed subnormal a-b waves and increased b-wave latency in LA3.0, whereas the second case showed a marked decrease in b-wave amplitude in the cone–rod response. Both patients showed an increase in b-wave latency in DA3.0. The same apparent features associated with CDSRR have been noted in previous studies in patients with a late formation of a b-wave with a supernormal amplitude in the rod–cone ERG response [14,21]. Essentially, when a high stimulus is needed to activate a response after transduction, an increase in b-wave amplitude will be obtained [4], while impaired phototransduction can directly affect a-wave changes in the ERG [5]. Due to the young age of our patients, it was not possible to observe ERG progression in both patients, whereas some studies have reported that full-field ERG parameters do not deteriorate and do not present age-related progress [23]. In general, the possibility of reliable classification can be determined by familiarity with the CDSRR characterization and complete observation of disease progression [23,24]. Furthermore, the delay in peak time between the a-wave and b-wave and the significant increase in b-wave amplitude between the two dimmest stimuli in ERG recordings may be key features for assessing the natural progression of *KCNV2* retinopathy [25].

*KCNV2* retinopathy is characterized by genetic variants leading to a loss of function, yet the underlying mechanisms contributing to photoreceptor cell degeneration in CDSRR remain unclear. SD-OCT analyses have indicated alterations in the expression and co-expression of *KCNV2* at the junction of the inner and outer segments of photoreceptors. Additionally, patients diagnosed with CDSRR exhibit notable reductions in foveal depth, diminished cone density, and structural disruptions of cone photoreceptors. While the differential effects of *KCNV2* retinopathy on cone versus rod photoreceptors are not fully elucidated, the evidence suggests the preservation of inner retinal function, providing a basis for prospective therapeutic strategies aimed at restoring outer retinal functionality [26]. Furthermore, investigations utilizing Kv8.2 knockout (KO) mice have identified the presence of natural killer (NK) cells in the retinal context, proposing an inflammatory mechanism as a potential contributor to the photoreceptor cell damage observed in *KCNV2* retinopathy [27].

## 5. Conclusions

The analysis of the two case reports on *KCNV2* retinopathy underscores the phenotypic variability and progressive nature of this retinal condition associated with mutations in the *KCNV2* gene. Both cases illustrated distinct clinical presentations, highlighting the spectrum of symptoms from early-onset hypermetropia and slight exotropia to more advanced retinopathy characterized by electroretinogram (ERG) changes and fundoscopic findings. Depending on the changes in the *KCNV2* gene and age, clinical manifestations may vary from patient to patient. In the first patient with a de novo variant at position 1109dup, the characteristic clinical course differs from the second patient, with the exception of similarities in visual acuity impairment and the type of strabismus. Overall, these case studies contribute to the growing body of literature on *KCNV2* retinopathy and highlight the critical need for further research to elucidate its pathophysiology and therapeutic options.

## Data Availability

The original contributions presented in the study are included in the article, further inquiries can be directed to the corresponding authors.
